# Strain Gauge Measuring System for Subsensory Micromotions Analysis as an Element of a Hybrid Human–Machine Interface

**DOI:** 10.3390/s22239146

**Published:** 2022-11-25

**Authors:** Olga Bureneva, Nikolay Safyannikov

**Affiliations:** Department of Computer Science and Engineering, Saint Petersburg Electrotechnical University “LETI”, 197022 Saint Petersburg, Russia

**Keywords:** strain gauge measuring system, involuntary motor acts, voluntary isometric efforts, analog signal filtering, analog signal averaging, visual feedback, sensory-motor functions, force measurement

## Abstract

The human central nervous system is the integrative basis for the functioning of the organism. The basis of such integration is provided by the fact that the same neurons are involved in various sets of sensory, cognitive, and motor functions. Therefore, the analysis of one set of integrative system components makes it possible to draw conclusions about the state and efficiency of the other components. Thus, to evaluate a person’s cognitive properties, we can assess their involuntary motor acts, i.e., a person’s subsensory reactions. To measure the parameters of involuntary motor acts, we have developed a strain gauge measuring system. This system provides measurement and estimation of the parameters of involuntary movements against the background of voluntary isometric efforts. The article presents the architecture of the system and shows the organization of the primary signal processing in analog form, in particular the separation of the signal taken from the strain-gauge sensor into frequency and smoothly varying components by averaging and subtracting the analog signals. This transfer to analog form simplifies the implementation of the digital part of the measuring system and allowed for minimizing the response time of the system while displaying the isometric forces in the visual feedback channel. The article describes the realization of the system elements and shows the results of its experimental research.

## 1. Introduction

Recently, a large number of studies have been devoted to understanding the role of the human central nervous system (CNS) as an integrative basis for the functioning of the organism. The CNS integrates, groups, and compares various sensory, motor, and cognitive signals, and makes decisions based on them. This integration is possible because the same neurons are involved in a diverse set of sensory, cognitive, and motor functions [1,2]. Therefore, analysis of one of the components of the integrative system makes it possible to draw conclusions about the state and efficiency of the other elements. The connection between motor and cognitive functions is also confirmed by the development of the “double task” technique [3,4]. This technique assumes the simultaneous performance of a motor task or maintenance of a steady posture and solution of cognitive tasks (internal counting, tasks on short-term memory and attention, divergent tasks). Dual tasks with simultaneous involvement of motor and cognitive functions are associated with specific characteristics of brain activity and make it possible to evaluate brain resources both in normal conditions and in the case of brain damage or age-related changes [5]. The process of solving dual tasks reveals the main neurophysiological mechanisms of bilateral transfer and the interference of cognitive and motor functions [6,7,8]. It has now been established that when performing complex dual tasks, more effective coordination of cognitive processes takes place, providing enhanced opportunities for successful cognitive functioning. Thus, assessment of human sensory-motor functions is important for building human–machine systems and for controlling behavior, functional states, and decision making related to cognitive processes.

The most informative in terms of assessing a person’s cognitive properties is the estimation of involuntary motor acts (subsensory reactions), i.e., those which are not controlled by a person. Such a motor act is tremor defined as a rhythmic involuntary oscillatory movement of at least one functional area of the body. There are two main types of tremors: pathological and physiological [9]. Pathological tremor is caused by various disorders in the central or peripheral parts of the nervous system and can indicate diseases of the central nervous system [10], including cognitive disorders [11]. Under some conditions, a healthy person may have a physiological tremor, which is most often invisible to the unaided eye because its amplitude is insignificant. However, such a tremor has definite frequencies. Under certain conditions, such as physical activity, under the influence of adrenaline produced because of fear or excitement, as well as when taking medications, the physiological tremor increases and becomes visible. Regardless of the cause of the physiological tremor, its characteristics are unchanged: the physiological tremor appears as a low-amplitude tremor with the frequency of 8–12 Hz [12,13]. The pathological tremor has other frequency characteristics depending on the disease [14]. Moreover, tremor frequency is one of the markers of the disorder used for the diagnosis.

Currently, various instrumental methods for measuring tremor parameters are actively being developed and used in clinical practice: mechanography [15], electromyography [16], methods based on video registration [17], methods involving the use of various sensors (force registration sensors, accelerometers, capacitive sensors, sensors recording changes in magnetic field during movement, etc.) [18,19,20], and special wearable devices [21,22]. A detailed comparative review of the sensors allowing for the estimation of the tremor is given in [23]. Combined methods have also been developed that measure various physical parameters simultaneously [24]. However, the listed methods focus on the analysis of visible tremor. Such tremor is pathological in most cases. Physiological tremor usually does not manifest itself externally; therefore, known methods of detection and determination of tremor parameters using accelerometers, motion sensors, magnetic field sensors cannot be used to measure physiological tremor parameters. Observing physiological tremor is difficult because its manifestation must be stimulated, for example, by physical exertion, temperature changes, or by inducing a person into a stress state. One way of observing physiological tremor is based on its monitoring against the background of isometric effort: the tremor occurs when muscles are strongly tensed without realization of movement. It may occur when clenching the hand into a fist or holding a heavy object [25]. Visual biofeedback is used to monitor CNS control signals during force generation [26].

## 2. Materials and Methods

We used the method for analyzing physiological tremor formed against the background of isometric force. For this purpose, we measured the voluntary isometric force controlled by the person by means of visual feedback and extracted involuntary fluctuations in this force. This method is based on the notions of the hierarchical multilevel organization of the central (motor) nervous system with the ring control based on sensory corrections. Using the model of neural structures of the segmental level of muscle contraction control, it was revealed that cyclic activity is supported in the ring structure. Its frequency is determined by the length of the ascending and descending pathways, the rate of excitation conduction in them, and the delay for signal processing in each functional brain region. It has been proven that of all types of analyzed movements, the parameters of the isometric effort controlled by a person give the most complete information on the integral activity of the central structures of the motor system.

During movement, muscles can realize eccentric (stretching) or concentric (contraction) actions; the third type of muscle action is isometric: muscles tense but do not change their length. Isometric load is performed in a static position and while it is held the involuntary movements caused by subsensory oscillatory muscle contractions occur. The obtained characteristics of the force formed by a person will contain information both about its constant component (isometric force) and its frequency component (tremor). The task of primary processing of information about the effort is to separate the constant and frequency components and obtain their amplitude–frequency characteristics.

### 2.1. Testing Procedure

**Figure 1:**
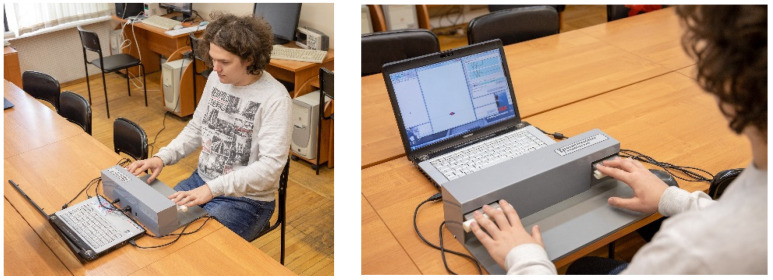
Testing procedure.

To measure human subsensory reactions, we used the following procedure [27]. A person being tested sits at a table, on which a monitor and a strain gauge measuring system with two supporting elements are placed. To take measurements, the person presses the measuring system’s supporting elements with their fingers and the arms stretched forward, and the amount of force is monitored using two marks on the monitor screen realizing this biofeedback, as shown in Figure 1. The marks on the screen move along the vertical axis in proportion to the applied force: one mark corresponds to the force of the right hand and the other to the left hand.

The task of the tested person is to keep the marks on the screen at the same set level. Under the conditions of holding isometric force, involuntary oscillations in the hands occur. These oscillations are not controlled by the person; their characteristics are indicators of the brain state. The testing protocol is approved by the ethical committee of the Human Brain Institute of the Russian Academy of Sciences named after N.P. Bekhtereva.

### 2.2. Strain Gauge Measuring System

**Figure 2:**
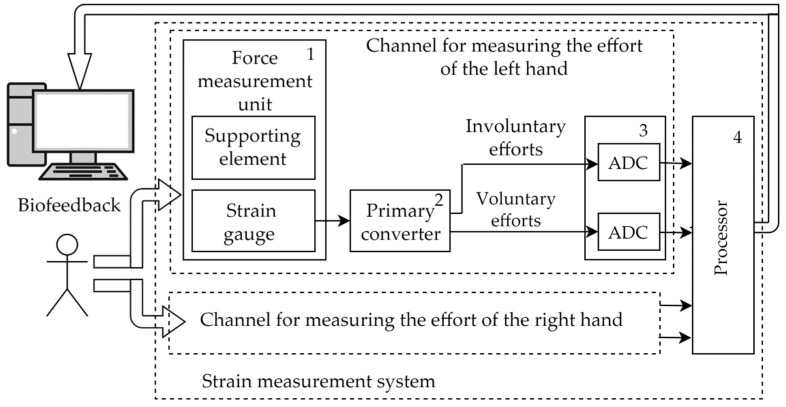
Structure of the strain gauge measuring system.

Figure 2 shows the strain gauge measuring system and its place in the human–machine interface. The measuring system, which implements the described procedure for measuring human subsensory reactions, contains two identical measuring channels that provide measurements of right- and left-hand forces. Each measuring channel includes:

1. A force measurement unit;
2. A primary converter;
3. Analog-to-digital converters;
4. A processor.

A person acts on the supporting elements; the strain-gauge generates an electrical signal proportional to the pressure. The primary converter performs signal filtering and divides the signal into components corresponding to arbitrary (isometric) force and involuntary force (tremor). Analog-to-digital converters (ADC) digitize the measurement results. The measurement results in digital form are transmitted to the system processor; it forms frames in a specified format and transmits them to a personal computer using the USB interface.

Digital codes corresponding to periodic signal components (involuntary efforts) are used for mathematical processing by various methods [28,29,30] in order to evaluate the state of the human brain. Digital codes corresponding to isometric effort are used to move the marks on the monitor; according to the movement of these marks, a person corrects his/her actions on the supporting elements of the system.

## 3. Development of the Measurement System

### 3.1. Force Measurement Unit

The force measurement unit converts the isometric force applied by the fingers of the person being tested to the supporting elements into an electric signal corresponding to the value of the force. The strain-gauges are used to measure the applied force; their function is based on the phenomenon of the tensoresistive effect—the property of solid materials to change the active resistance of conductors at their various mechanical deformations.

The resistance of the strain-gauges changes under the influence of the physical forces generated by the person under test. These changes are very small and produce slight current variations. Since the force measurement range is large and reaches several kilograms, the changes in the output signal can be less than one percent of the initial value. A bridge circuit should be used to convert such small changes. Additionally, a means should be provided to reduce the temperature dependence of the sensor output signal.

There are several classes of sensors in which, in addition to the sensor element, a measuring bridge is implemented and temperature compensation is provided. A built-in bridge circuit of strain gauges connection provides sufficiently high sensitivity and thermal stability. The requirements of the sensors used to measure the isometric force generated by human hands are determined by the characteristics of human impact:

- The maximum magnitude of the voluntary force sufficient for research for different groups of persons should be 10 kg.
- The amplitude of the isometric force oscillation does not exceed 2% of the voluntary force.
- The forces produced by involuntary arm oscillation are mostly periodic; significant oscillations are detected in the range of 0–16 Hz.

Along with the periodic components of the effort, the signals also contain aperiodic components of involuntary hand oscillations. These components are characterized by high rates of rise and fall of the fronts of individual impulses; however, due to their limited number, they do not have a noticeable influence on the spectral distribution of the effort. Nevertheless, their presence is evident when considering changes in the force on the time diagrams. Thus, the final choice of the sensor was made taking into account the parameters of registered signals and mechanical and design features. The S-shaped Self-Temperature Compensated (STC) strain-gauges (manufacturer—Celton company [31]) with the following technical parameters were used:

- Standard capacities—25 kg;
- Type of force transmitted—Bi-directional (tension/compression);
- Rated output (RO)—3.0 mV/V;
- Zero balance—±1.0% of RO;
- Rated output tolerance—±0.25% of RO.

**Figure 3:**
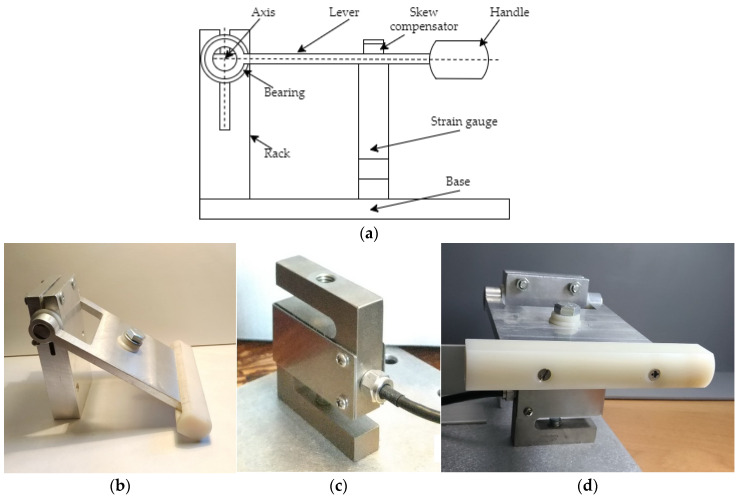
Force measurement unit: (**a**) schematic design, (**b**) side view, (**c**) S-shaped strain gauge, (**d**) handle side view with sensor installed.

The dimensions of the selected sensor determined the design of the support elements for the test person’s hands. Figure 3 shows the design of the force measurement unit. The unit consists of: base, rack, bearing, axis, lever bar, handle made of insulating material, skew compensator, strain gauge.

The strain gauge is mounted under the lever bar and is rigidly attached to the base of the force measurement unit. The force applied to the lever bar is transmitted to the sensor with a screw installed through the bar and the skew compensator. The bar is connected to the axle that runs through two bearings secured in the rack. The location of the strain gauge under the lever bar has been chosen to suit the measuring range and allows the maximum signal to be received when human force is applied.

### 3.2. Primary Converter

**Figure 4:**
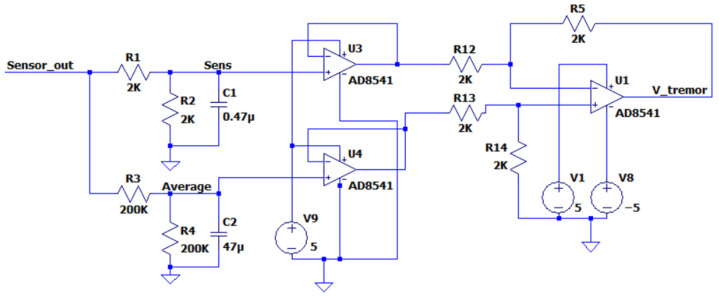
Circuit of the primary conversion of a strain gauge output signal.

The signal at the strain gauge output is quite small and contains noise. Before digitizing, it should be amplified and filtered. The sensitive element of the strain gauge is connected by a bridge circuit, so we took the signal rationing scheme [32] as the basis, having modified it to solve the problem of separating the signal into smoothly varying and frequency components. Figure 4 represents the scheme of the primary signal processing.

The circuit contains the following elements: R1, R2, and C1 are elements for noise filtering in the signal Sensor_out, taken from the output of the strain gauge. The time constant for the selected values of resistance and capacitance ensures the suppression of the components of the signal Sensor_out with a frequency above F = 2 kHz. R3, R4, and C2 are elements for averaging the signal Sensor_out. The values of the resistors and the capacitor provide averaging of the signal for 1 s.

**Figure 5:**
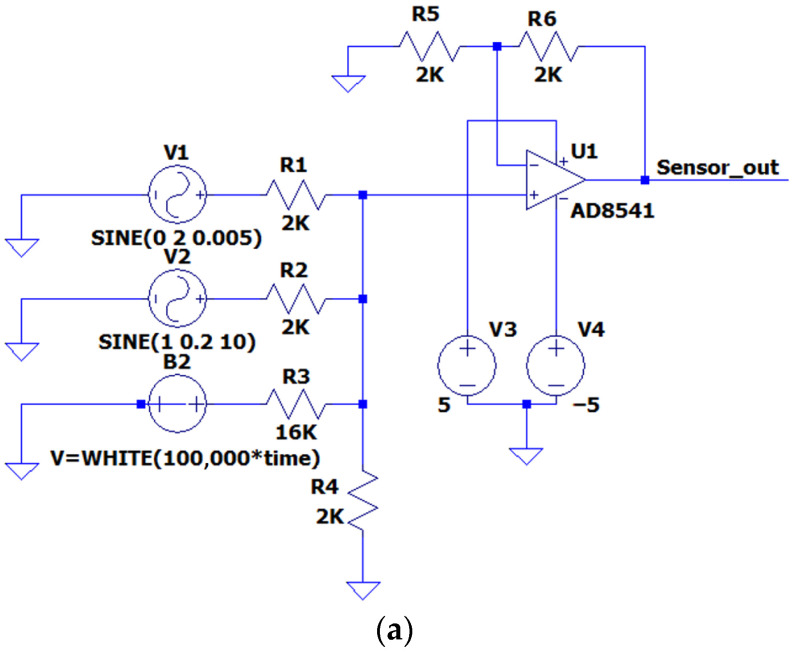

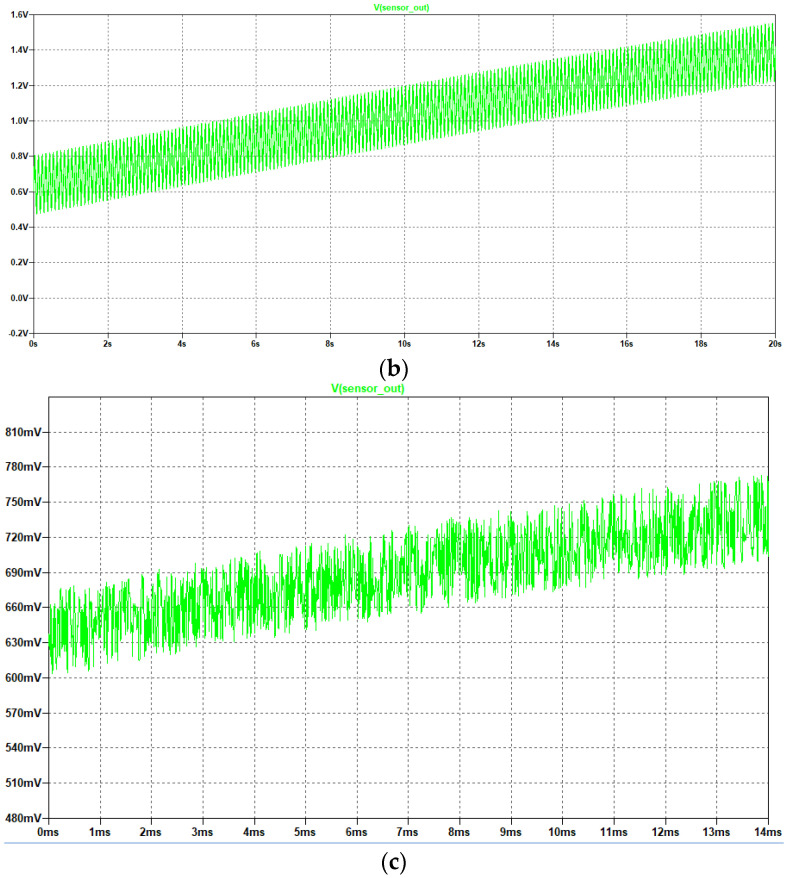
Test signal generation: (**a**) circuit for the test signal generation, (**b**,**c**) generated output signal at different display scales (Sensor_out).

To test the proposed circuit, the model simulating the signal taken from the strain gauge was developed (Figure 5). The circuit forms a signal by summing the following signals:

- Sine with an amplitude of 2 V and a frequency of 0.005 Hz (provides a smooth signal change, simulates isometric force);
- Sine with amplitude of 0.2 V and frequency of 10 Hz (provides generation of the frequency component of the signal, simulating a tremor);
- White noise.

**Figure 6:**
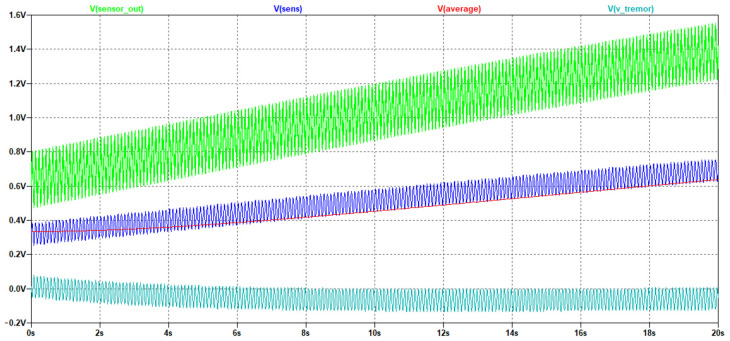
Simulation result.

Simulation of the circuit operation was performed in the LTSpice system [33]. Figure 6 shows the simulation result. A smoothly increasing noisy periodic signal, Sensor_out (green), was used as the input of the primary conversion circuit. As a result of filtering and scaling the signal, Sens (blue) was obtained—the signal value from the sensor cleared from the noise over 2K Hz. The Sens signal values are two times lower than the Sensor_out signal due to the use of the voltage divider.

The average signal (red) is the average value of the Sens signal. The result of subtracting the average values from the Sens signal values is the signal V_tremor (light blue), which characterizes subsensory human reactions. The analysis of our simulation results showed that the proposed circuit correctly performs signal transformations. Its main disadvantage is the presence of a transient process when the output signal fluctuates. After the finishing of the transient process, the characteristics of the test signal at the output of the circuit do not change. This fact should be taken into account in further digital signal processing.

Based on the parameters of the primary converter output signal and the frequency characteristics of involuntary human movements, the requirements for the ADC are determined. To register and display the frequency and time properties of the strain gauge sensor signals when a human hand is acting on them, the following parameters must be provided: for signal spectrum analysis—30 Hz; for the analysis of the signal shape on the time characteristics—150 Hz. According to Nyquist’s theorem [34], the transmission frequency of discrete signal values must be higher than twice the upper frequency of the spectrum of the analyzed signal. That is, the ADC conversion frequency should not be less than 300 Hz. The estimation of the resolving power can be calculated on the basis of the noise parameters of the devices connected directly to the sensor. Typical values of the noise parameters are given in the specifications and are in the range of (80 ÷ 600) nV. The number of ADC bits is defined as follows: N = log_2_(dynamic range/peak-to-peak noise); numerically, it is as follows: N = log_2_(2V/600 nV) = 21.2.

To implement the analog-to-digital conversion, the ADS1246 chip was chosen. This is a delta-sigma ADC with a bit depth of 24 bits. In the developed system, it was set to continuous conversion mode with a frequency of 1000 sps, which corresponds to the single-transformation duration being within 1 ms. The used ADC ADS1246 allows higher conversion frequency, but when the data output rate was increased to 2000 sps, the lower bits of the output code fluctuated. The duration of the first conversion differs slightly from the duration of the second and subsequent conversions—the first conversion is performed about 1 ms longer, which does not violate the requirements for subsequent measurements. All ADC settings are performed by writing specific codes for the ADC registers via the SPI interface. The same interface is also used to transfer digitized data to the system processor. A C8051F320 microcontroller from Silicon Laboratories Inc. is used as a processor. It provides ADC settings, receives digitized measurement results, and transmits them to the PC via USB 2.0.

The primary signal processing circuIt was mounted on operational amplifiers and discrete elements. The assembled measuring system was connected to a personal computer, and measurements were performed.

## 4. Results

**Figure 7:**
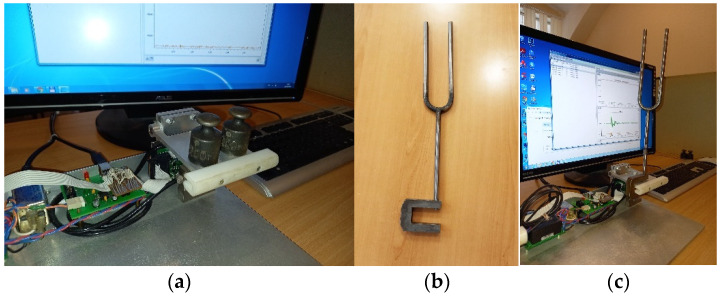
Testing of the strain gauge measuring system: (**a**) checking the measurement of the constant component of the sensor signal using a set of weights; (**b**) tuning fork; (**c**) checking the measurement of the frequency component of the sensor signal using a tuning fork.

The developed system was built and tested. For this purpose, we developed special software, which receives the measurement results via USB interface, displays them graphically, and saves them in csv format. The testing process is shown in Figure 7.

A set of weights was used to test the system when measuring constant forces. Different weights were placed on the supporting elements, and measurements were carried out; the duration of the measurements was 3 s.

Table 1 shows a fragment of the measurement results obtained within 1 s. The columns present the following values: i—number of measurements; time—time stamps in seconds. Moreover, the codes obtained at the output of the analog-to-digital converter are as follows: LConst and RConst—codes characterizing the constant components of the signals in the channels for the measurement of the left- and right-hand forces, respectively; LTrem and RTrem—codes characterizing the frequency components of the signals.

The average values of the constant and frequency components of the signals are calculated using the following formula:

$$\;\bar{X}\;=\frac{1}{n}\overset{n}{\underset{i=1}{\sum }}X_{i}$$

and have the following values: $\bar{\mathrm{LConst}}$ = 13.7; $\bar{\mathrm{RConst}}$= 284.698; $\bar{\mathrm{LTrem}}$ = −2.0255; $\bar{\mathrm{RTrem}}$ = −1.09796.

Deviations from the described above average values were calculated as follows:

$$X\text{\_}d=\sqrt{\frac{1}{n}\overset{n}{\underset{i=1}{\sum }}{\left(X_{i}-\;\bar{X}\;\right)}^{2}}$$

where LConst_d = 0.240858; RConst_d = 0.360159; LTrem_d= 0.139287; and RTrem_d = 0.323387. Similar results were obtained when measured on other scales. Thus, a high stability of the measurement results is shown.

**Figure 8:**
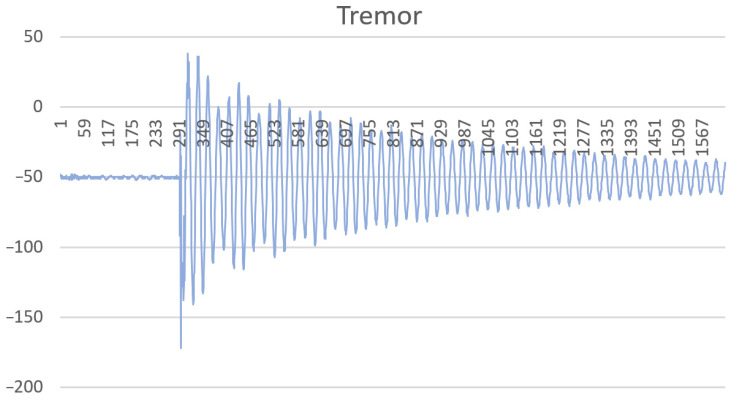
The fading harmonic oscillation obtained as a result of the activation of the tuning fork.

To control the measurements of the periodic component of the signal, a tuning fork was made to form oscillations with a frequency of 150 Hz. During the testing of the system, the tuning fork was placed on the supporting element of the system, as shown in Figure 7. Excitation of the tuning fork was performed by striking it with a rubber hammer. Figure 8 shows the measurement result in graphical form. The measurement result is represented by a time series. The values of the series correspond to the code taken from the ADC and are proportional to the amplitude of the signal. The marks on the *x*-axis are the number of the samples in the time series. It is a graph of a slowly fading harmonic oscillation. The distances between the peaks correspond to time stamps with a difference of about 6 ms, which corresponds to a frequency of 150 Hz.

**Figure 9:**
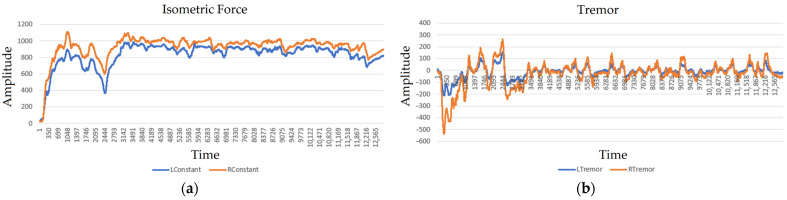

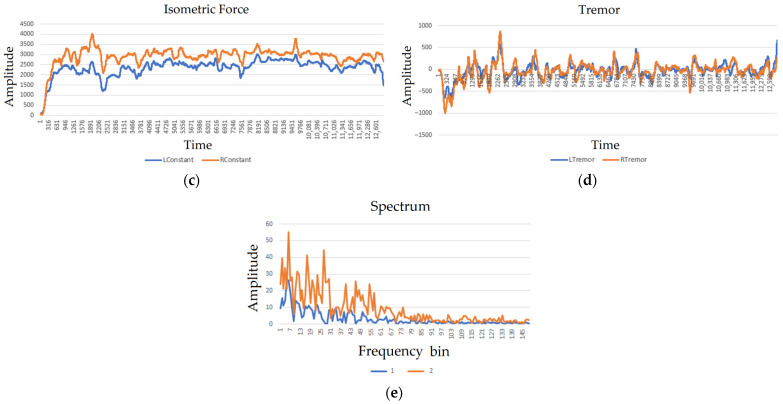
Results of measuring subsensory reactions of a healthy person: (**a**) left (LConstant)- and right (RConstant)-hand isometric effort (Test 1); (**b**) left (LTremor)- and right (RTremor)-hand subsensory reactions (Test 1); (**c**) left (LConstant)- and right (RConstant)-hand isometric effort (Test 2); (**d**) left (LTremor)- and right (RTremor)-hand subsensory reactions (Test 2); (**e**) spectral characteristics of hand subsensory reactions for tests 1 and 2.

Figure 9 shows the result of measuring the isometric component of force and tremor in a healthy person in two tests differing in the value of applied isometric force. The values of the series in Figure 9a–d correspond to the code taken from the ADC and are proportional to the amplitude of the signal. The marks on the *x*-axis are the number of the samples in the time series. The difference between time samples corresponds to approximately 0.00102 s, which is reflected in Table 1.

The results presented correspond to two measurements. In the first case, the subject was asked to hold the force on the mark corresponding to 1000 g, in the second—3000 g. When holding a small force, the fingers lightly act on the sensing elements, and the parameters of fluctuations in the involuntary force component are insignificant.

When sustaining a great effort, involuntary oscillations become less regular; there appear moments of increased amplitude and frequency. Prolonged holding of the maximum force makes it impossible to hold the marks accurately on the monitor screen. Synchronous corrective flashes in the left and right hand appear. At this point, the person performs force adjustment to the specified tracking level.

The increase in the force is accompanied by an increase in the amplitudes of the spectral response in the range of 2.5–12 Hz characterized by a further smooth decrease. The components of the spectrum in the region above 2.5 Hz increase with the increase in force. The force holding is accompanied by increased activity in the ranges of 4–6 and 10–12 Hz. Thus, the correlation between the voluntary isometric force and the frequency characteristics of tremor is shown.

## 5. Conclusions

This paper presents a system enabling the estimation of the parameters of human subsensory reactions. The reactions arise in response to the isometric forces generated. The novelty of the work consists of the fact that the good interpretability of the results is determined not by the accuracy of the sensors used and specialized hardware of signal processing but by a principally new way of analytics organization, that is, the extraction of involuntary movement components against the background of isometric effort. In a person in a stable psychophysiological state, amplitude–frequency characteristics of involuntary oscillations are directly connected with isometric effort. In a psychophysiological disturbed person under stress or under the influence of some substances, as well as in patients with CNS disorders, these correlates are impaired. We have implemented a system that allows us to evaluate correlations between voluntary and involuntary acts.

In contrast to the implementation of the strain gauge system [26], where the main measured force parameters were transferred to the computer and processed programmatically, in this development we focused on the hardware implementation of signal conversion. In the system, preprocessing of measured signals is performed in analog form. Due to the use of analog filters and analog averaging, it was possible to minimize the system response time when displaying isometric forces in the visual feedback channel. Transferring the calculations (signal averaging and separation of the frequency component against the background of a smoothly changing signal) to the preprocessing area allowed us to simplify mathematical data processing. During the testing of human subsensory reactions, physically interpretable results were obtained.

## Figures and Tables

**Table 1:** Measurement results with constant pressure on the right-hand support element of the strain gauge measuring system.

i	Time	LConst	LTrem	RConst	RTrem
1	0	13	−2	284	−1
2	0.00102	14	−2	285	−1
3	0.002041	13	−2	285	0
4	0.003061	13	−2	284	−2
5	0.004082	15	−3	285	0
6	0.005102	13	−1	283	1
7	0.006122	14	−2	284	−3
8	0.007143	14	−2	287	−2
9	0.008163	14	−3	284	0
0	0.009184	13	−2	284	−3
11	0.010204	15	−3	287	−2
12	0.011224	14	−2	285	0
13	0.012245	13	−1	283	−1
14	0.013265	13	−2	286	−1
15	0.014286	13	−2	284	−1
…	…				
979	0.997959	13	−2	284	−1
980	0.99898	14	−2	285	−2
Average values		13.7	−2.0255	284.698	−1.09796
Deviations		0.240858	0.139287	0.360159	0.323387

## Data Availability

Not applicable.
